# Inhibition of Cyclooxygenase-2 Prevents Chronic and Recurrent Cystitis

**DOI:** 10.1016/j.ebiom.2014.10.011

**Published:** 2014-10-24

**Authors:** Thomas J. Hannan, Pacita L. Roberts, Terrence E. Riehl, Sjoerd van der Post, Jana M. Binkley, Drew J. Schwartz, Hiroyuki Miyoshi, Matthias Mack, Reto A. Schwendener, Thomas M. Hooton, Thaddeus S. Stappenbeck, Gunnar C. Hansson, William F. Stenson, Marco Colonna, Ann E. Stapleton, Scott J. Hultgren

**Affiliations:** a Department of Pathology & Immunology, Washington University School of Medicine, St. Louis, MO 63110, USA; b Department of Molecular Microbiology and Center for Women's Infectious Disease Research, Washington University School of Medicine, St. Louis, MO 63110, USA; c Division of Allergy & Infectious Diseases, Department of Medicine, University of Washington, Seattle, WA 98195, USA; d Division of Gastroenterology, Department of Medicine, Washington University School of Medicine, St. Louis, MO 63110, USA; e Department of Medical Biochemistry, University of Gothenburg, 40530 Gothenburg, Sweden; f Department of Internal Medicine, University of Regensburg, 93053 Regensburg, Germany; g Institute of Molecular Cancer Research, University of Zurich, 8091 Zurich, Switzerland; h Division of Infectious Diseases, Department of Medicine, University of Miami Miller School of Medicine, Miami, FL 33136, USA

**Keywords:** ASB, asymptomatic bacteriuria, CD, clusters of differentiation, COX, cyclooxygenase, G-CSF or CSF3, granulocyte colony-stimulating factor, GRO-α or CXCL1, growth-regulated alpha protein, IL-8 or CXCL8, interleukin-8, IBC, intracellular bacterial community, M-CSF or CSF1, macrophage colony-stimulating factor, MAb, monoclonal antibody, MCP-1 or CCL2, monocyte chemotactic protein 1, NSAID, non-steroidal anti-inflammatory drug, rUTI, recurrent urinary tract infection, UTI, urinary tract infection, UPEC, uropathogenic *E. coli*, Immunomodulatory therapy, Mucosal immunology, Immunopathology, Uropathogenic *E. coli*, UPEC, COX-2, Urinary tract infection, UTI, recurrent infection, Chronic infection

## Abstract

The spread of multidrug-resistant microorganisms globally has created an urgent need for novel therapeutic strategies to combat urinary tract infections (UTIs). Immunomodulatory therapy may provide benefit, as treatment of mice with dexamethasone during acute UTI improved outcome by reducing the development of chronic cystitis, which predisposes to recurrent infection. Here we discovered soluble biomarkers engaged in myeloid cell development and chemotaxis that were predictive of future UTI recurrence when elevated in the sera of young women with UTI. Translation of these findings revealed that temperance of the neutrophil response early during UTI, and specifically disruption of bladder epithelial transmigration of neutrophils by inhibition of cyclooxygenase-2, protected mice against chronic and recurrent cystitis. Further, proteomics identified bladder epithelial remodeling consequent to chronic infection that enhances sensitivity to neutrophil damage. Thus, cyclooxygenase-2 expression during acute UTI is a critical molecular trigger determining disease outcome and drugs targeting cyclooxygenase-2 could prevent recurrent UTI.

## Highlights

• Discovery of serum biomarkers of recurrent UTI (rUTI) in women implicates enhanced myeloid cell responses.
• Inhibition of cyclooxygenase-2 disrupts bladder epithelial transmigration by neutrophils and protects against rUTI in mice.
• Bladder epithelial remodeling that occurs consequent to chronic infection enhances mucosal sensitivity to neutrophil damage.

## Graphical abstract

**Figure:**
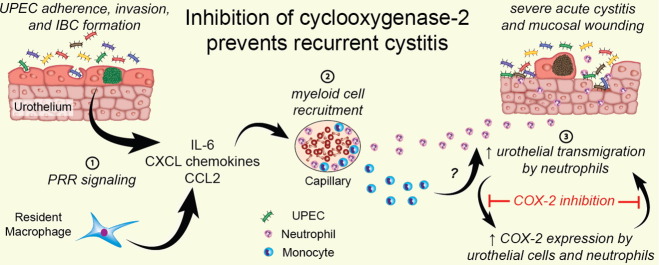

## 1 Introduction

The rapid and global dissemination of antibiotic-resistant bacteria has resulted in dwindling therapeutic options for many infectious diseases, highlighting the urgent need for new therapies (Gupta and Bhadelia, 2014). One common infection for which effective therapies are needed is urinary tract infections (UTIs), which, as assessed in 2007 account for 10.5 million outpatient and emergency room visits per year in the United States alone (Foxman, 2014, Schappert and Rechtsteiner, 2011). UTI is a significant cause of morbidity in women throughout their lifespan and in infant boys and older men, with serious sequelae including frequent recurrences, pyelonephritis with sepsis and renal damage in young children. Further, UTI has been associated with pre-term birth, and complications of frequent antimicrobial use, including high-level antibiotic resistance and *Clostridium difficile* colitis. Uropathogenic *Escherichia coli* (UPEC) cause approximately 85% of community-acquired UTI and virulent multi-drug resistant UPEC clones have recently emerged worldwide (Gupta and Bhadelia, 2014). This increases the cost and length of treatments and threatens to lead to untreatable disease, unless strategies for new effective therapies and treatments are developed. Although cystitis can be self-limiting, in the absence of effective antibiotic therapy, studies have shown that up to 60% of women experience bacteriuria lasting months after initial infection often despite improvement of symptoms (Ferry et al., 2004, Mabeck, 1972).

Murine models of UTI in young naïve mice have elucidated critical details of acute UPEC pathogenesis, involving the invasion of UPEC into bladder epithelial (urothelial) cells (Hannan et al., 2012, Brumbaugh and Mobley, 2012). Internalized UPEC are able to avoid a TLR4-mediated exocytic process (Song et al., 2009) and escape into the host cell cytoplasm, where they replicate into biofilm-like intracellular bacterial communities (IBCs) (Justice et al., 2004, Anderson et al., 2003). IBCs are routinely observed in urine cytology of individuals presenting with UTI, supporting the validity of their importance in pathogenesis and the ability of the mouse model to recapitulate human disease (Rosen et al., 2007, Robino et al., 2013, Robino et al., 2014). This process allows UPEC to establish infection and persist in the face of a stringent population bottleneck (Hannan et al., 2012, Schwartz et al., 2011) caused by the host's acute multi-prong defense: including secretion of cytokines (Duell et al., 2012, Ingersoll et al., 2008, Ragnarsdottir et al., 2011), activation and infiltration of immune cells (Haraoka et al., 1999, Schiwon et al., 2014, Chan and St John, 2013), and exfoliation of epithelial cells (Mulvey et al., 1998, Dhakal and Mulvey, 2012). Exactly how these host responses act in a coordinated fashion to clear infection, how a multitude of UPEC virulence factors act to promote infection, and how bacterial and host factors interact to determine disease outcome and susceptibility to recurrent UTI (rUTI) are poorly understood.

There are two main outcomes of UPEC bladder infection in naïve mice: i) sterilization of the urine within days of acute infection with or without the establishment of a quiescent intracellular reservoir (Mysorekar and Hultgren, 2006, Mulvey et al., 2001), or ii) persistent high titer bacteriuria and chronic high titer bladder infection with chronic bladder inflammation (chronic bacterial cystitis) that lasts for the lifetime of the animal if not cleared by appropriate antibiotics (Hannan et al., 2010). Which of these outcomes occurs after UPEC infection in C3H/HeN mice is determined within the first 24 h post-inoculation (hpi) and depends on the severity of the host's acute inflammatory response (Hannan et al., 2010). Specifically, severe pyuria and bladder inflammation with elevated serum interleukin-5 (IL-5) and serum and urine IL-6, the neutrophil chemokine CXCL1, and granulocyte colony-stimulating factor (G-CSF or CSF3) at 24 hpi are predictive of chronic infection. Whether chronic cystitis in mice is analogous to an untreated clinical chronic symptomatic UTI or an acute symptomatic UTI that resolves into asymptomatic bacteriuria (ASB) is not clear, but in contrast to immunodeficient mouse models of ASB (Ragnarsdottir et al., 2011) chronic cystitis in immunocompetent mice results from ongoing extracellular bacterial replication on the inflamed bladder mucosa in the face of a robust neutrophil response. This chronic bladder inflammation manifests as both lymphonodular hyperplasia in the bladder submucosa and urothelial hyperplasia, with a lack of uroplakin expression, a marker for terminal differentiation, in superficial facet cells (Hannan et al., 2010). Similar histological findings have been observed in humans suffering persistent bacteriuria and recurrent UTI (Schlager et al., 2011, Hansson et al., 1990). Significantly, chronic bladder inflammation in mice appears to cause mucosal remodeling that renders the bladder more susceptible to UTI upon further bacterial challenge weeks after resolution of the primary infection with antibiotic therapy, suggesting that this provides a clinically relevant model for rUTI (Hannan et al., 2010).

Interestingly, transient immunosuppression of mice by a single treatment with the synthetic glucocorticoid dexamethasone prior to infection reduces the severity of acute inflammation and protects against chronic infection. Based on the complexities of UTI pathogenesis, the rapid emergence of multi-drug resistant UPEC strains, and the key role the host response plays in the disease course and outcome, interest is growing in the development of treatments that facilitate bacterial clearance by modulating the host immune system. In this study, we hypothesized that a dexamethasone-sensitive host–pathogen checkpoint exists early during the pathogenesis of UTI that determines host susceptibility to chronic and recurrent infection. We identified serum biomarkers associated with sensitivity to rUTI in clinical samples from women with cystitis and subsequently investigated the mechanistic basis behind these biomarkers using mouse models of severe acute, chronic, and recurrent cystitis. These studies revealed that excessive neutrophil infiltration of the urothelium and cyclooxygenase-2 (COX-2) dependent inflammation are critical components of the acute host-pathogen checkpoint that both exacerbated and prolonged infection. These findings provide a therapeutic rationale for targeting COX-2 in the prevention and treatment of rUTI.

## 2 Materials and Methods

### 2.1 Clinical Study Population

The study was conducted at the Hall Health Primary Care Center, an outpatient clinic at the University of Washington, Seattle, WA. Women were eligible if they were aged 18–49 years, in good general health, and had typical symptoms of acute cystitis (dysuria, frequency, and urgency) for < 7 days. Women were not eligible if they had temperature ≥ 100 °F or flank pain or tenderness, nausea or vomiting, chronic illness requiring medical supervision (e.g., diabetes mellitus), known anatomic or functional abnormalities of the urinary tract, urinary catheterization, UTI within the past month, or were pregnant or planning pregnancy in the next 3 months or not contracepting. The Human Subjects Review Committee of the University of Washington approved the study, and all subjects gave written informed consent.

### 2.2 Clinical Study Procedures

Flyers, newspaper ads and discussions with local clinicians were the main methods of recruitment, and subjects were enrolled as soon as possible after contacting study personnel. At the study visit, subjects underwent a history and physical examination, including a vaginal examination, and an interview using a standardized questionnaire. Subjects were instructed to obtain a clean-catch midstream urine specimen for culture and to provide a peripheral blood sample. Urine samples for culture were refrigerated and transported to the laboratory within 24 h of collection. Blood was collected in tubes without anti-coagulant, allowed to clot at room temperature for 1–2 h, and serum was isolated by centrifugation and stored at − 80 °C until analysis.

Women were treated with trimethoprim–sulfamethoxazole double strength (DS) twice daily for 3 days unless they were allergic or intolerant to this agent or were infected with a uropathogen known to be resistant to this agent in which case they were treated with ciprofloxacin 250 mg twice daily for 3 days or nitrofurantoin (Macrobid®) 100 mg twice daily for 7 days. Participants were asked to return to the clinic if their symptoms did not resolve or if they recurred during the study at which time they provided a midstream urine specimen and then treated using the same treatment protocol as at enrollment. Participants were followed for 3 months.

### 2.3 Clinical Laboratory Procedures

Methods for collecting urine specimens and isolating, identifying, and quantifying uropathogens have been previously described (Stamm et al., 1982, Anon., 1999). All uropathogens (Gram-negative rods, enterococci, Group B streptococci and *Staphylococcus saprophyticus*) were identified and quantified to 10^2^ cfu/ml.

### 2.4 Sample Selection & Serum Cytokine Analysis

The presence of 48 human cytokines was analyzed in serum specimens by a Luminex-based multiplex cytometric bead array platform (Bioplex, Bio-Rad, Hercules, CA). Serum specimens for testing were drawn from the pool of enrollment UTIs where the sole causative uropathogen was *E. coli*. Forty-one women with an *E. coli* recurrent UTI were identified, and a comparison group was randomly culled from the women in whom there was no recurrent UTI within the three-month follow-up period. The cytokines analyzed were interleukin-1α (IL-1α), IL-1β, IL-1ra, IL-2, IL-2Rα, IL-3, IL-4, IL-5, IL-6, IL-7, IL-8, IL-9, IL-10, IL-12 (p40), IL-12 (p70), IL-13, IL-15, IL-16, IL-17, IL-18, basic fibroblast growth factor (FGF-basic), eotaxin, cutaneous T-cell attracting chemokines (CTACK or CCL27), gamma interferon (IFN-γ), granulocyte colony-stimulating factor (G-CSF or CSF3), granulocyte–macrophage colony-stimulating factor (GM-CSF or CSF2), growth-regulated alpha protein (GRO-α or CXCL1), hepatocyte growth factor (HGF), IFN-α2, interferon gamma-induced protein 10 (IP-10 or CXCL10), leukemia inhibitory factor (LIF), monocyte chemotactic protein 1 (MCP-1 or CCL2), MCP-3 (CCL7), macrophage colony-stimulating factor (M-CSF or CSF1), macrophage migration inhibitory factor (MIF), monokine induced by gamma interferon (MIG or CXCL9), macrophage inflammatory protein 1α (MIP-1α or CCL3), MIP-1β (CCL4), nerve growth factor (β-NGF), platelet-derived growth factor (PDGF-BB), Regulated upon Activation Normal T-cell Expressed and Secreted (RANTES or CCL5), stem cell factor (SCF), stem cell growth factor beta (SCGF-β), stromal cell-derived factor 1 alpha (SDF-1α), tumor necrosis factor α (TNF-α), TNF-β, TNF-related apoptosis-inducing ligand (TRAIL), and vascular endothelial growth factor (VEGF). Individual samples were run in duplicate and the mean values used in all analyses.

### 2.5 Animal Studies Ethics Statement

All animal experimentation was conducted following the National Institutes of Health guidelines for housing and care of laboratory animals and performed in accordance with Institutional regulations after pertinent review and approval by the Institutional Animal Care and Use Committee at Washington University School of Medicine.

### 2.6 Bacterial Strains and Cultivation

The UPEC strain primarily used in this study was a kanamycin-resistant derivative of the human cystitis isolate, UTI89 (Schilling et al., 2001): UTI89 *att_HK022_*::*COM-GFP* (UTI89 Kan^R^) (Wright et al., 2005). For UPEC challenge of previously infected mice, we used a spectinomycin-resistant UTI89 derivative: *attλ*::*PSSH10-1* (spectinomycin-resistant, Spc^R^) (Wright et al., 2005). Bacteria were routinely cultured in lysogeny broth (LB).

### 2.7 Mouse Infections

C3H/HeN mice were obtained from Harlan Sprague Dawley, Inc. (Indianapolis, IN). Bacterial strains were inoculated into 20 ml of LB directly from freezer stock, grown statically at 37 °C overnight, and sub-cultured 1:1000 into 20 ml of fresh LB and again grown statically at 37 °C for 18 h. These cultures were spun at room temperature for 10 min at 3000 ×*g*, resuspended in 10 ml phosphate-buffered saline, pH = 7.4 (PBS), and diluted to approximately 2–4 × 10^8^ colony forming units (cfu)/ml (OD_600_ = 0.35). 50 μl of this suspension (~ 1–2 × 10^7^ cfu) or one concentrated 10-fold (~ 1–2 × 10^8^ cfu) was inoculated into the bladders of 7–8 week old female mice by transurethral catheterization.

### 2.8 Treatments

Drugs and antibodies were administered as described in the main text. Indomethacin, SC-236 and SC-560 were purchased from Sigma (St. Louis, MO), solvated in 1% Tween 80 in PBS, and 100 μl of each drug suspension or buffer alone per 20 mg body weight was administered by oral gavage. Clodronate and PBS liposomes were manufactured as previously described (Seiler et al., 1997, Zeisberger et al., 2006). Rat anti-C-C chemokine receptor type 2 (CCR2) (clone MC-21) was generated as previously described (Mack et al., 2001, Bruhl et al., 2007). Rat anti-lymphocyte antigen 6G (Ly6G) (clone 1AG) and rat IgG2a and IgG2b isotype controls were purchased from Bio X Cell (West Lebanon, NH). All antibodies were diluted in tissue culture grade sterile Dulbecco's PBS and 200 μl of each antibody solution were injected intraperitoneally as indicated.

### 2.9 Urine Collection, Bacterial Titering, and Urine Sediment Analysis

Urines were collected at 1, 3, 7, 10, and 14 dpi, and then weekly thereafter by applying suprapubic pressure with proper restraint and collecting the urine stream in sterile 1.5 ml Eppendorf tubes. Urines were then serially diluted in PBS and 10 μl total of each dilution was spotted onto LB and LB with 25 μg/ml kanamycin (LB/Kan25) agar plates. Urine sediments were obtained by cytocentrifuging 80 μl of a 1:10 dilution of the collected urine onto poly-l-lysine-coated glass slides and stained as described (Rosen et al., 2007). Stained urine sediments were examined by light microscopy on an Olympus BX51 light microscope (Olympus America), and the average number of polymorphonuclear leukocytes (PMN) per 400× magnification field (hpf) calculated from counting 5 fields. A semi-quantitative scoring system of 0–5 was modified from an earlier study to facilitate analysis: 0, less than 1 PMN/hpf; 1, 1–5 PMN/hpf; 2, 6–10 PMN/hpf; 3, 11–20 PMN/hpf, 4, 21–40 PMN/hpf, and 5, > 40 PMN/hpf (Hannan et al., 2010).

### 2.10 Tissue Bacterial Titer Determinations

Bladders and kidneys were aseptically harvested at indicated time points and homogenized in PBS. Homogenates were then serially diluted and spotted as described above onto LB and LB/Kan25 agar plates.

### 2.11 Flow Cytometry

Single-cell bladder suspensions were made from minced bladder tissues subjected to collagenase IV/DNase I digestion for 90 min at 37 °C and then passed through a 40-μm filter, and cells were washed as described previously (Ingersoll et al., 2008, Guiton et al., 2013). Staining of surface markers was performed in FcR block with fluorochrome-conjugated monoclonal antibodies (MAbs). Cells were counterstained with propidium iodide (PI) prior to flow cytometry, and only live (PI-low) cells were included in the analysis. To specifically characterize the myeloid cell infiltrates, combinations of MAbs specific for the following surface markers were chosen as described in the results section: CD11b, F4/80, Ly6G, Ly6C, CCR2, and CD45. All antibodies were from BD Pharmingen, eBioscience, Miltenyi or R&D Systems. Sample data were acquired on either a FACSCalibur or an LSRFortessa flow cytometer (BD Biosciences), and data were analyzed using FlowJo software (version 7.6.4). The relative proportion of cellular infiltrates in each bladder was calculated as a percentage of live cells.

### 2.12 Histopathology and Immunofluorescence

Tissues were fixed in either 10% neutral buffered formalin or methacarn (60% methanol, 30% chloroform, 10% glacial acetic acid). Fixed tissues were embedded in paraffin, sectioned, and some slides were stained with hematoxylin and eosin (H&E). Bladder inflammatory scores were determined blindly on two serial H&E-stained sections of each bladder and an average score calculated, as previously described (Hopkins et al., 1998). For immunofluorescence microscopy, bladder sections were deparaffinized, hydrated and blocked in 1% BSA, 0.3% Triton X-100 in PBS. After incubation with primary and secondary antibodies and associated washes, slides were stained with bis-benzimide (Sigma). Primary antibodies used were specific for COX-1 (goat polyclonal, sc-1754, Santa Cruz Biotechnologies), COX-2 (mouse monoclonal, 610204, BD Transduction Laboratories) and Ly6G (rat monoclonal, 127610, Biolegend). Stained tissues were examined by epifluorescence microscopy on a ZEISS Axioskop 2 MOT Plus microscope.

### 2.13 Proteomics & Pathway Analysis

Bladders were isolated from mock, resolved and sensitized mice (n = 3) four weeks after antibiotic therapy, snap frozen in liquid nitrogen and stored at − 80 °C. Fat and debris were removed from bladders and the tissue was cut open into a flat sheet. The tissue was incubated in 0.1% ethylenediaminetetraacetic acid (EDTA), 10 mM HEPES pH 7.4 in PBS at 37 °C for 1 h while gently shaken. Epithelial cells were released by shaking the tissue samples for 5 min at 700 rpm using an orbital shaker after which the remaining tissue was removed from the tube and fixed in methacarn for analysis of isolation efficiency. Cells were pelleted by centrifugation for 5 min at 500 rpm and directly resolved in 500 μl 2 M NaCl, 1 mM EDTA in 10 mM HEPES–NaOH pH 7.4 containing complete protease inhibitor cocktail (Roche), and homogenized by tip probe sonication (UltraTurbax T8, IKA, Staufen, Germany) at maximum for 30 s. Membrane proteins were enriched as described (Lu et al., 2009), with all centrifugation steps performed at 100,000 *g* for 20 min. Pellets were solubilized in 0.1 M dithiothreitol (DTT), 4% sodium dodecyl sulfate (SDS), 0.5% polyethylene glycol (PEG) 20,000 in Tris/HCl pH 7.6 and heated at 90 °C till completely resolved and clarified by centrifugation for 5 min at maximum rpm (Eppendorf 5415R, Germany). Supernatants were transferred to 30,000 kDa cut-off filters (NanoSep, Pall) and mixed with 200 μl 8 M urea in 100 mM Tris/HCl pH 8.5. Proteins were digested according to the filter-aided sample preparation method (Wisniewski et al., 2009) using two-step digestion with endoprotease Lys-C (Wako) followed by trypsin (Promega). Recovered peptides were differentially isotopically labeled using dimethyl (CH^3^, CHD^2^ and 13CD^3^) according to the on-column method as described (Boersema et al., 2009). Labeled peptides were eluted from the solid phase extraction (SPE) columns with 5 mM ammonium acetate, 0.5% formic acid in 95% acetonitrile and the peptide concentration was determined based on the absorbance at 280 nm. Samples were mixed at equal ratio and separated into 8 fractions using zwitterionic hydrophilic interaction liquid chromatography (ZIC-HILIC) (SeQuant, Umeå, Sweden) as described earlier (van der Post et al., 2013). Fractions were dried under vacuum and resolved in 15 μl 0.2% TFA prior to mass spectrometry analysis.

Peptide analyses were performed by online nanoflow liquid chromatography tandem mass spectrometry (nLC–MS/MS) using an Easy-nLC 1000 system (Thermo) coupled to a Q-Exactive mass spectrometer (Thermo). Briefly, samples were loaded on to a Kasil fritted in-house packed column (50 μm inner diameter, 200 mm length) with 1.8 μm C18 material (Reprosil-AQ Pur, Dr. Maisch) connected to a steel emitter (Proxeon) using a zero dead volume connector. Chromatography was performed at 200 nl/min using 0.2% formic acid in water (mobile phase A) and 0.2% formic acid, 80% acetonitrile in water (mobile phase B) and peptides were eluted over an 80 min gradient from 5% to 35% B. The mass spectrometer was operated in data dependent mode switching automatically between MS and MS/MS mode. Full scans were obtained between 350 and 1600 m/z followed by 12 MS/MS scans on the top multiple charged precursor ions using higher-energy collisional dissociation (HCD) at a normalized collision energy of 30%, which were then excluded from fragmentation for 45 s.

Spectral data were processed using MaxQuant version 1.3.0.5 (Cox and Mann, 2008) supported by the Andromeda search engine (Cox et al., 2011). Searches were performed against all mouse proteins found in TrEMBL and Swiss-Prot (2013_04, 42921 entries) concatenated with a reversed database, and the false discovery rate (FDR) was set to > 1% for both peptide and protein identification. First search precursor mass tolerance was set to 20 ppm for recalibration, 6 ppm for the main search and 20 ppm for MS/MS spectra. Trypsin was set as enzyme allowing for 2 miss-cleavages except when KR was followed by P. Fixed modifications were set for carbamidomethyl (C) and variable for oxidation (M) and acetylation (protein N-terminal). Match between run options within a 2 min time window was enabled for constitutive fractions and only proteins identified in two out of three biological replicates were considered. Quantification was performed using the light dimethyl label for mock, intermediate for resolved and heavy for sensitized mice and ratios were calculated and expressed as log2 values. Probabilities were calculated based on significance B (Cox and Mann, 2008) with FDR estimation using the Benjamini–Hochberg procedure. All downstream bioinformatics analyses were done using R (www.r-project.org). Protein–protein interaction data were retrieved from STRING (version 8) database for the significantly (*P* < 0.05) regulated proteins and further network analyzes were performed using Cytoscape (Shannon et al., 2003, Jensen et al., 2009). Interaction networks were combined with gene ontology (GO) annotation for both molecular function and biological process to identify functional clusters using the ClueGO plugin (Bindea et al., 2009).

### 2.14 RNA Extraction and Quantitative RT-PCR

RNA was extracted from UTI89 infected mouse bladders at indicated time points as well as from PBS mock-infected mouse bladders using the RNeasy Plus Mini kit (QIAGEN) and reverse-transcribed with the iScript Reverse Transcription Supermix (Biorad). For quantitative reverse transcription polymerase chain reaction (qRT-PCR), 1 μl of 12.5 ng/μl cDNA was used with intron-spanning primer pairs specific to *mPtgs1* (F: 5′-cctctttccaggagctcaca-3′, R: 5′-tcgatgtcaccgtacagctc-3′) and *mPtgs2* (F: 5′-gatgctcttccgagctgtg-3′, R: 5′-ggattggaacagcaaggattt-3′) (designed using the Roche Universal Probe Library Assay Design tool) and the iQ SYBR Green supermix according to the manufacturer's instructions (Bio-Rad). Expression values were normalized to 18S (F: 5′-cggctaccacatccaaggaa-3′, R: 5′-gctggaattaccgcggct-3′) expression levels and the fold difference relative to mock-infected bladders was determined by the 2^− ΔΔCt^ method (Pfaffl, 2001). Each sample was run in triplicate and average C_T_ values calculated.

### 2.15 In situ Hybridization

In situ hybridization studies were performed as previously described (Manieri et al., 2012). Briefly, digoxigenin-labeled antisense RNA probes were synthesized from a cDNA clone for Ptgs2 (clone ID: 30059181, Thermo Fisher Scientific) with T7 RNA polymerase (New England Biolabs, Ipswich, MA) and DIG RNA Labeling Mix (Roche, Indianapolis, IN) and purified with NucAway Spin Columns (Life Technologies, Carlsbad, CA). Deparaffinized and protease K-treated sections were hybridized with RNA probes and incubated with anti-digoxigenin alkaline phosphatase (AP)-conjugated antigen binding fragments (Fab) (Roche). Specific signals were visualized with nitro blue tetrazolium chloride (NBT) and 5-bromo-4-chloro-3-indolyl phosphate (BCIP) (Roche) and sections were counterstained with methyl green.

### 2.16 UPEC Challenge Infections

At 28 dpi with 10^8^ cfu of UTI89 Kan^R^, mice with persistent high-titer (> 10^4^ cfu/ml) bacteriuria throughout infection were treated with trimethoprim and sulfamethoxazole in the drinking water daily for 10 days at concentrations of 54 and 270 μg/ml, respectively (Schilling et al., 2002). During this time, longitudinal urinalysis was continued weekly to confirm clearance of bacteriuria. Four weeks after the initiation of antibiotic therapy these sensitized mice were challenged with 10^8^ cfu of UTI89 Spc^R^ 30 min after drug treatment. Longitudinal urinalysis was then performed as for the primary infection, except now triplicate plating onto LB, LB/Kan25 and MacConkey with 50 μg/ml spectinomycin (McC/Spc50) agar to identify mice with persistent bacteriuria and the responsible strain. Mice were sacrificed 4 weeks after challenge and tissue titers determined as above, triplicate plating onto LB, LB/Kan25, and LB/Spc50 agar.

### 2.17 Statistical Analysis

Statistical analyses were performed using GraphPad Prism and InStat (GraphPad Software) and significance was defined by attaining *P* values < 0.05, in two-tailed tests.

## 3 Results

### 3.1 Identification of Candidate Serum Biomarkers of Recurrent UTI in Women

To assess whether serum biomarkers could identify patients susceptible to rUTI, we tested for the presence of 48 cytokines and growth factors in the banked enrollment (V_0_) sera from a clinical study of sexually active, premenopausal women who presented with acute UTI with UPEC and were followed for three months to determine recurrence (Table 1). Levels of serum cytokines and growth factors involved in monocyte and macrophage development (IL-3 and M-CSF/CSF1), chemotaxis (MCP-1/CCL2), and differentiation (M-CSF/CSF1) and neutrophil development (IL-3) and chemotaxis (GROα/CXCL1 and IL-8/CXCL8) were increased in patients who developed rUTI compared to those that did not suffer recurrence (Table 2 and Table S1). For four of these cytokines, this elevation was most pronounced in the subset of patients for whom the enrollment UTI was their first reported lifetime UTI (Table 2). For example median levels of M-CSF were increased two-fold in first time UTI patients developing rUTI compared to those who did not develop rUTI during the study (*P* = 0.04, Mann–Whitney U test). These patient data suggest that higher levels of systemic inflammatory markers during acute cystitis in previously naïve individuals is associated with rUTI, in agreement with what was previously found in mice (Hannan et al., 2010).

### 3.2 Temperance of Bladder Neutrophil Recruitment Protects Against Chronic Cystitis

Based upon the patient serum cytokine data, we investigated the role of neutrophils, monocytes and macrophages in our animal model of chronic cystitis. Using flow cytometry, we found that CD11b^+^ myeloid cells, and particularly neutrophils (Ly6G^+^ Ly6C^int^ F4/80^−^) and inflammatory monocytes (Ly6G^−^ Ly6C^hi^ F4/80^int/lo^), are the most abundant innate immune cells recruited to the severely inflamed bladder at 24 hpi in UPEC-infected C3H/HeN mice (Fig. S1A and Fig. S1B). In contrast, the relative abundance of resident macrophages (CD11b^+^ Ly6G^−^ Ly6C^−^ F4/80^hi^) did not change with infection. Furthermore, increased relative abundances of both neutrophils and inflammatory monocytes correlated significantly with increased bladder weight (*P* < 0.05 for each, *r*^2^ = 0.67 and 0.60, respectively, Spearman's rank-order correlation) (Fig. S1C). To investigate the role of these myeloid cell populations in cystitis, we first tested whether neutrophils were required to control UPEC infection by treating mice with a high dose of the neutrophil-depleting anti-Ly6G MAb (clone 1A8) (Daley et al., 2008). In agreement with a previous study that used the anti-granulocyte differentiation antigen 1 (Gr-1) (clone RB6-8C5) MAb (Haraoka et al., 1999), which has since been shown to also deplete Ly6C^+^ inflammatory monocytes, we found that neutrophil depletion resulted in all mice developing severe infection and chronic cystitis (Fig. S1D). Chronic cystitis in mice is here defined as the development of persistent high titer bacteriuria (> 10^4^ cfu/ml at each time point over 4 weeks of infection) and high titer bladder bacterial burdens (> 10^4^ cfu per bladder) at sacrifice 4 weeks post-infection (wpi).

**Fig. 1:**
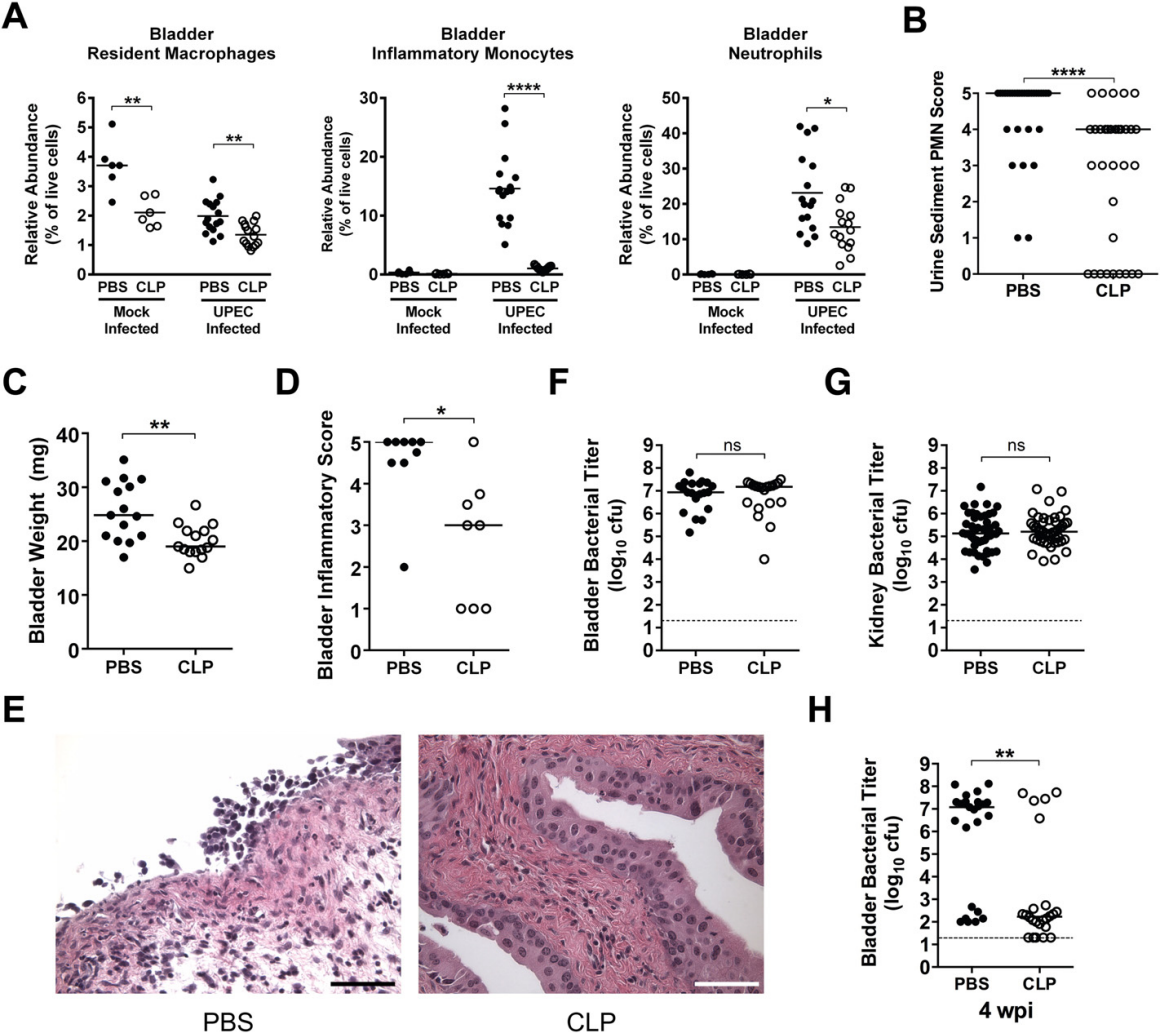
Clodronate liposome treatment reduces acute bladder inflammation and protects C3H/HeN mice from chronic cystitis. Mice were either treated with 2 mg of PBS liposomes (PBS, closed circles) or 2 mg of clodronate liposomes (CLP, open circles) intraperitoneally 8 h prior to intravesical inoculation with 10^8^ cfu of the UPEC strain UTI89 or PBS (mock) bladder infection and tissues harvested 24 hpi for analysis unless otherwise indicated. (A) The relative abundances of the indicated cell lineages were determined by flow cytometry. (B) The abundance of neutrophils in the urine sediment was determined semi-quantitatively by microscopic examination. (C) Bladder wet weights were measured. (D–E) A subset of bladders was scored for inflammation. Images from bladders with the median inflammatory score are shown in panel E, bars approximate 50 μm. (F) Bladder and (G) kidney titers were determined. (H) Bladder titers were determined 28 dpi. In graphs, data points shown represent actual values for each individual mouse and data are combined from 2 to 5 independent experiments; bars indicate median values and dashed lines indicate the limit of detection; all statistics shown used the Mann–Whitney U two-tailed test; ns: not significant, * *P* < 0.05, **P < 0.01, ***P < 0.001, ****P < 0.0001. See also Fig. S1.

We then depleted monocytes and tissue macrophages using clodronate liposomes (CLP) (Seiler et al., 1997, Zeisberger et al., 2006), to test whether these myeloid cells impact the incidence of chronic cystitis. Compared to control PBS liposomes, CLP treatment 8 h prior to intravesical inoculation with PBS significantly reduced bladder resident macrophage populations by 43% (Fig. 1A). In contrast, CLP treatment eliminated the F4/80^+^ CD11b^lo/−^ red pulp macrophage population in the spleen (data not shown), suggesting that bladder resident macrophages are somewhat protected from the effects of CLP treatment. Upon UPEC infection, we found that CLP treatment not only eliminated the recruitment of inflammatory monocytes, resulting in near background (mock-infected) levels, but also significantly reduced the bladder resident macrophage and recruited neutrophil populations (Fig. 1A) and significantly reduced the severity of pyuria (Fig. 1B) and gross bladder inflammation at 24 hpi (Fig. 1C–E). Intriguingly, overall acute bacterial burdens at this time point were similar in the bladder and kidneys irrespective of monocyte and macrophage depletion (Fig. 1F and G). Yet, the reduction in acute bladder inflammation and mucosal damage observed at 24 hpi correlated with a reduction in the incidence of chronic cystitis at 4 wpi (Fig. 1H; 72% (18/25) vs. 21% (5/24), *P* < 0.001, Fisher's exact test), a protective effect that is similar to what we previously reported with dexamethasone treatment (Hannan et al., 2010).

**Fig. 2:**
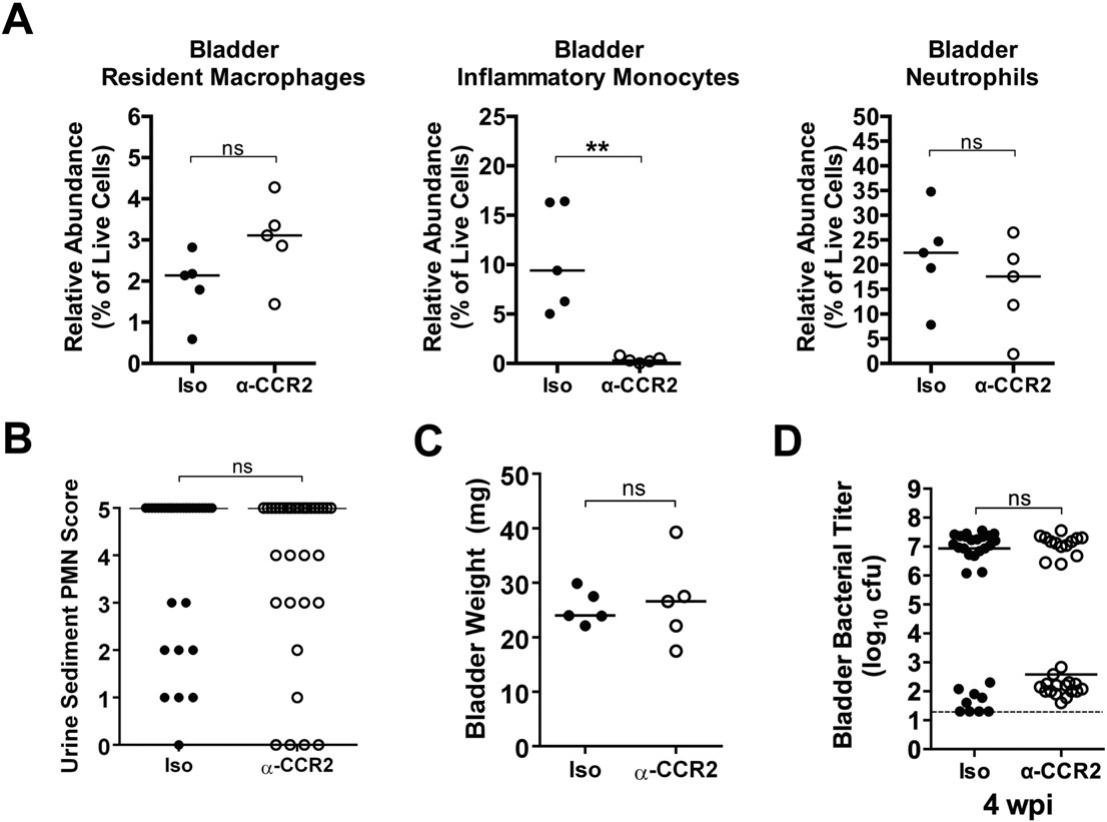
Depletion of inflammatory monocytes does not reduce pyuria or protect against chronic cystitis. Mice were either treated with 20 μg of the MC-21 anti-CCR2 monoclonal antibody (α-CCR2, open circles) or 20 μg of isotype control antibody (Iso, closed circles) intraperitoneally 2 h prior to intravesical inoculation with 10^8^ cfu of the UPEC strain UTI89. (A) The relative abundances of the indicated cell lineages were determined by flow cytometry 24 hpi. (B) The abundance of neutrophils in the urine sediment at 24 hpi was determined semi-quantitatively by microscopic examination. (C) Bladder wet weights were measured 24 hpi. (D) Bladder titers were determined 28 dpi. In graphs, data points shown represent actual values for each individual mouse and data are combined from 1 to 4 independent experiments; bars indicate median values and dashed lines indicate the limit of detection; all statistics shown used the Mann–Whitney U two-tailed test; ns: not significant, **P < 0.01.

It was recently suggested that Ly6C^+^ inflammatory monocytes recruited to the urinary bladder during acute UTI are critical for “licensing” resident macrophages to induce bladder epithelial transmigration by neutrophils (Schiwon et al., 2014). To test whether depletion of inflammatory monocytes alone was sufficient to reduce pyuria and protect against chronic cystitis in our model we treated mice with MC-21, an anti-CCR2 monoclonal antibody that depletes inflammatory monocytes (Mack et al., 2001, Bruhl et al., 2007). CCR2 is the receptor for monocyte chemokines such as CCL2 and CCL7 and bone marrow egress of monocytes and their recruitment to the urinary bladder has been reported to be dependent upon CCR2 signaling (Engel et al., 2006). We found that about half of inflammatory monocytes in the bladder retain CCR2 on their cell surface at 24 hpi (data not shown). Treatment with MC-21 eliminated the recruitment of inflammatory monocytes (CD11b^+^ F4/80^int^ Ly6C^+^) to the bladder, but did not affect neutrophil recruitment or resident macrophage numbers (Fig. 2A). Unlike CLP treatment, pretreatment with MC-21 did not significantly affect bladder weights (Fig. 2B) or the level of pyuria at 24 hpi compared to isotype treated control mice (Fig. 2C). There was a trend towards a decreased incidence of chronic cystitis in MC-21 compared to isotype control treated mice; however, the difference was not significant (Fig. 2D, 70% (21/30) vs. 45% (13/29), *P* = 0.07, Fisher's exact test). The lack of effect of MC-21 compared to CLP treatment suggests that the inflammatory response of bladder resident macrophages is able to compensate for the loss of inflammatory monocyte recruitment.

**Fig. 3:**
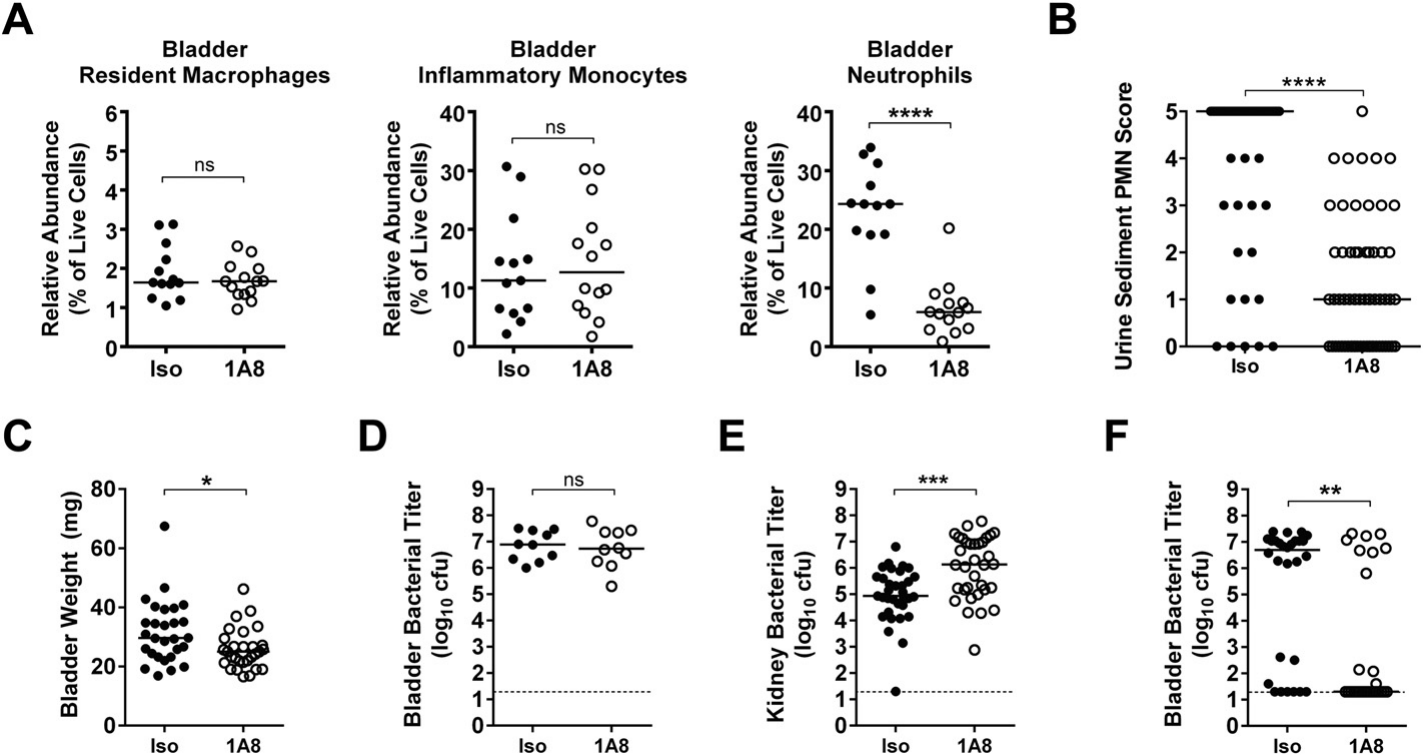
Partial depletion of neutrophils is sufficient to moderate acute bladder inflammation and protect C3H/HeN mice from chronic cystitis. Mice were treated with either 10 μg of the 1A8 anti-Ly6G monoclonal antibody (1A8, open circles) or 10 μg of isotype control antibody (Iso, closed circles) intraperitoneally 24 h prior to intravesical inoculation with 10^8^ cfu of the UPEC strain UTI89. (A) The relative abundances of the indicated cell lineages were determined by flow cytometry at 24 hpi. (B) The abundance of neutrophils in the urine sediment was determined semi-quantitatively by microscopic examination at 24 hpi. (C) Bladder wet weights were determined 24 hpi. (D) Bladder and (E) kidney titers were determined at 24 hpi. (F) Bladder titers were determined 28 dpi. In graphs, data points shown represent actual values for each individual mouse and data are combined from 2 to 6 independent experiments; bars indicate median values and dashed lines indicate the limit of detection; all statistics shown used the Mann–Whitney U two-tailed test; ns: not significant, * *P* < 0.05, **P < 0.01, ***P < 0.001, ****P < 0.0001.

Our monocytic cell depletion experiments demonstrated a strong correlation between neutrophil recruitment to the bladder and subsequent urothelial transmigration into the lumen, as indicated by pyuria, and susceptibility to chronic cystitis. To directly test whether robust neutrophilic inflammation is required to develop severe acute and chronic infection, we treated mice with a very low dose (10 μg) of the 1A8 Ly6G-specific antibody 24 h prior to infection to partially reduce neutrophil levels (Brandes et al., 2013). This treatment reduced the median relative abundance of recruited bladder neutrophils by 75% with an associated reduction in the levels of pyuria and bladder inflammation at 24 hpi (Fig. 3A–C), as quantified by bladder weight (edema). Interestingly, partial neutrophil depletion did not alter the bladder bacterial burden at 24 hpi (Fig. 3D), though UPEC titers were increased in the kidney (Fig. 3E). Strikingly, the attenuation of neutrophilic inflammation reduced the incidence of chronic cystitis to levels similar to that observed with CLP treatment (Fig. 3F, 68% (19/28) vs. 27% (8/30), *P* < 0.01, Fisher's exact test). Thus, while neutrophils are required to prevent overwhelming bladder infection, excessive neutrophilic inflammation leads to bladder immunopathology and chronic cystitis.

### 3.3 Proteomics Identifies Urothelial Remodeling in Sensitized Mice

**Fig. 4:**
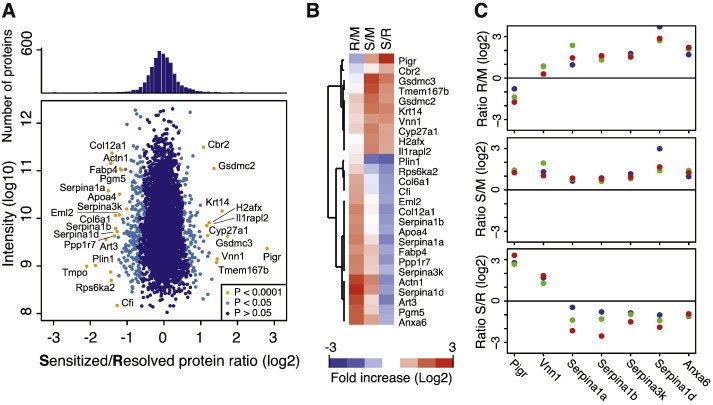
Proteomics identifies urothelial remodeling changes in sensitized mice that would render the bladder more susceptible to severe inflammation and neutrophil-mediated damage. (A) Quantitative proteomics analysis of convalescent bladder epithelia identified 4008 proteins. Average protein ratios for sensitized (S) versus resolved (R) bladders show the strongest enrichment or depletion for 27 proteins. (B) Heat map and tree of proteins with *P* value ≤ 0.0001 in the S/R comparison, also showing differences relative to age-matched mock (M) infected mice. (C) Data from a subset of proteins that are involved in inflammatory responses, showing the individual data points from each mouse in the three pairwise comparisons. See also Table S2. Proteomics identifies urothelial remodeling changes in sensitized mice that would render the bladder more susceptible to severe inflammation and neutrophil-mediated damage. (A) Quantitative proteomics analysis of convalescent bladder epithelia identified 4008 proteins. Average protein ratios for sensitized (S) versus resolved (R) bladders show the strongest enrichment or depletion for 27 proteins. (B) Heat map and tree of proteins with *P* value ≤ 0.0001 in the S/R comparison, also showing differences relative to age-matched mock (M) infected mice. (C) Data from a subset of proteins that are involved in inflammatory responses, showing the individual data points from each mouse in the three pairwise comparisons. See also Table S2.

C3H/HeN mice that develop chronic cystitis of > 2 weeks duration prior to antibiotic therapy become “sensitized” to severe recurrent chronic cystitis upon secondary bacterial challenge, whereas cage mates that spontaneously resolve the initial acute cystitis are resistant to secondary challenge (Hannan et al., 2010). We hypothesized that sensitized mice undergo remodeling changes as a consequence of chronic infection that leave them more prone to severe neutrophilic inflammation upon bacterial challenge. To investigate this, we obtained membrane-enriched fractions of urothelial cells isolated from mouse bladders 8 weeks after either PBS or UPEC inoculation and 4 weeks after treatment of antibiotics to resolve infection and compared the relative levels of proteins from three groups of mice (n = 3 mice per group): mock, resolved, and sensitized (as determined by longitudinal urinalysis) (Table S2). The comparison of bladder fractions from resolved and sensitized mice revealed differential expression of a number of proteins (Fig. 4). Proteins associated with gene ontology (GO) terms relating to innate immunity, acute inflammation, response to wounding, cell adhesion, and oxidative stress were particularly overrepresented. Of particular relevance to this work, we found that the relative amounts of many serpins, which are protease inhibitors that protect the epithelium from neutrophil-associated enzymes such as elastase and cathepsin G that can cause mucosal damage, were lower in sensitized mice compared to mice that spontaneously resolved infection. In addition, levels of vanin-1, a surface expressed enzyme that catalyzes the conversion of coenzyme A into pantothenic acid and cysteamine, a potent inducer of mucosal inflammation during oxidative stress, were significantly increased in sensitized mice. Lastly, levels of annexin VI, a known inhibitor of cytoplasmic phospholipase A2 (Cubells et al., 2008), which releases arachidonic acid from lipid membranes, the substrate of cyclooxygenases, was also lower in sensitized mice relative to resolved mice. The differences in levels of annexin VI raised the hypothesis that AA-derived mediators of acute inflammation may exacerbate acute neutrophilic inflammation in sensitized mice. This hypothesis is supported by our previous findings that the glucocorticoid analog dexamethasone, a potent suppressor of AA release, is protective against chronic cystitis (Hannan et al., 2010). Dexamethasone also specifically inhibits the inflammation-dependent expression of cyclooxygenase-2 (COX-2), which catalyzes the rate-limiting step in the conversion of AA to prostanoids, i.e. prostaglandins and thromboxanes (Rhen and Cidlowski, 2005). Thus, we investigated the role of the cyclooxygenases in the sensitization of mice to chronic cystitis and the therapeutic value of cyclooxygenase inhibitors.

### 3.4 COX-2 is Expressed by Neutrophils and Urothelial Cells During Severe Acute Cystitis

**Fig. 5:**
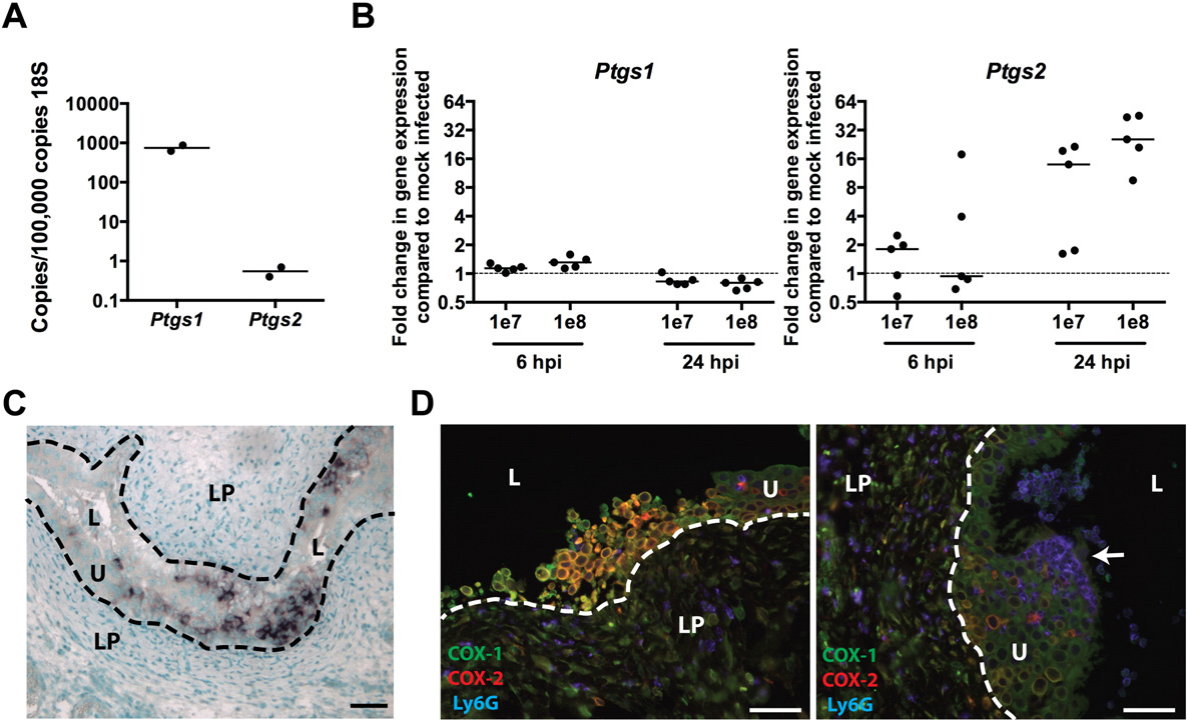
COX-2 is expressed by neutrophils and urothelial cells in severely infected bladders. (A–B) Urinary bladder COX-1 (*Ptgs1*) and COX-2 (*Ptgs2*) gene expression was determined by qRT-PCR in (A) mock PBS-infected bladders and (B) 6 and 24 h after intravesical inoculation with either 10^7^ or 10^8^ cfu of the UPEC strain UTI89. (C) COX-2 gene expression at 24 hpi was detected in UPEC-infected bladder tissue by in situ hybridization. (D) COX-1, COX-2, and Ly6G protein expression at 24 hpi in UPEC-infected bladders was detected by immunofluorescence microscopy of paraffin sections. In panels C–D, bars approximate 50 μm, L indicates bladder lumen, U indicates urothelium, LP indicates lamina propria, and dashed line denotes the approximate location of the urothelial basement membrane. The arrow indicates the presence of an IBC in the section. See also Fig. S2.

Cyclooxygenase (COX)-1 immunoreactivity has been detected in basal urothelial cells and mesenchymal cells of the uninfected bladder, but not COX-2 (de Jongh et al., 2009, de Jongh et al., 2007). However, upon UPEC infection COX-2 expression is induced in urinary particulates from patients with UTI (Wheeler et al., 2002), in bladder carcinoma cells in vitro (Chen et al., 2011), and in neutrophils during experimental cystitis in C3H/HeN mice (Poljakovic et al., 2001). In vivo, we found that the COX-2 transcript was barely detectable in uninfected mouse bladders by qRT-PCR, being present at 1000-fold lower transcript abundance levels than COX-1 (Fig. 5A). However, after UPEC infection, COX-2 mRNA expression was induced up to fifty-fold in the bladder by 24 hpi, whereas COX-1 expression did not change (Fig. 5B). Consistent with this, in situ hybridization showed no detectable COX-2 transcript at either 6 hpi or in mock-infected control bladders, whereas widespread and strong COX-2 mRNA staining was present in 2 of 5 UPEC-infected bladders at 24 hpi. COX-2 staining was confined to the urothelial layer in nests of basal and intermediate urothelial cells, as well as a few smaller cells with segmented nuclei, suggestive of neutrophils (Fig. 5C). Diffuse or scattered foci of weak urothelial COX-2 staining were observed in 2 of the remaining 3 bladders. Importantly, the presence of strong COX-2 expression in 2 of 5 bladders perfectly correlated with the presence of severe acute inflammation (indicated by bladder histology and bladder tissue weights greater than 30 mg), which is strongly predictive of chronic cystitis. Immunofluorescent antibody staining of bladder sections confirmed robust COX-2 expression by urothelial cells in those bladders with severe inflammation at 24 hpi (Fig. 5D and Fig. S2). In addition, non-epithelial cells both within the urothelium and the lamina propria stained mildly positive for COX-2, the vast majority of which were also Ly6G^+^, indicating that neutrophils are the predominant immune cells that express COX-2 during UPEC infection. Thus, severe bladder inflammation at 24 hpi correlates with the influx of COX-2 positive neutrophils and strong COX-2 expression by urothelial cells.

### 3.5 Inhibition of COX-2 Activity Protects Against Chronic and Recurrent Cystitis

**Fig. 6:**
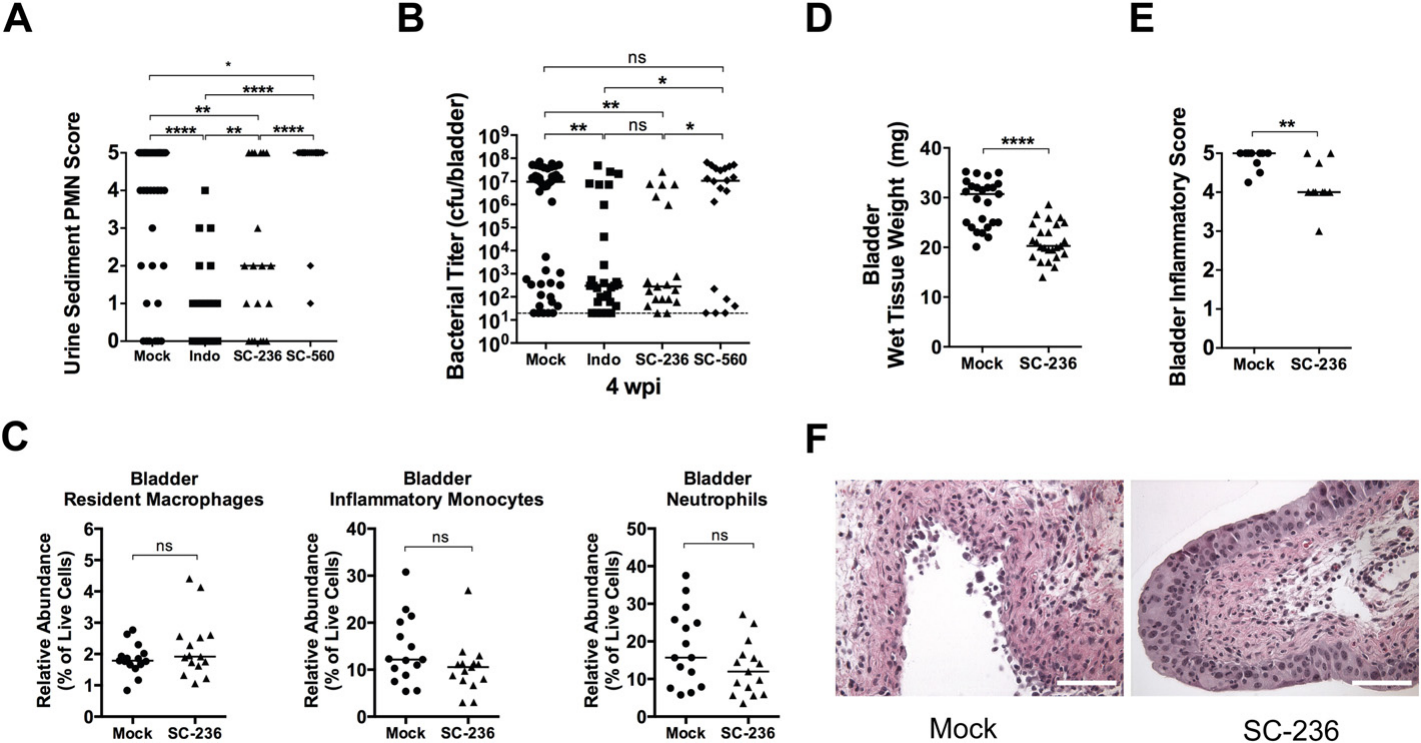
Inhibitors of COX-2 reduce the severity of acute bladder inflammation and protect against chronic cystitis in naïve mice. Mice were orally gavaged with 3.75 mg/kg of the NSAID indomethacin (Indo), 10 mg/kg of either a COX-2 specific inhibitor, (SC-236) or a COX-1 specific inhibitor (SC-560), or vehicle alone (Mock) 30 min prior to intravesical inoculation with 10^8^ cfu of the UPEC strain UTI89. Indomethacin and SC-560 treatments were repeated at 8 hpi, due to their short half-lives. All panels show data from 24 hpi unless otherwise indicated. (A) The abundance of neutrophils in the urine sediment was determined semi-quantitatively by microscopic examination. (B) Bladder titers were determined at 28 dpi. (C) The relative abundances of the indicated cell lineages were determined by flow cytometry. (D) Bladder wet weights were measured. (E–F) A subset of bladders was scored for bladder inflammation. Images from bladders with the median inflammatory score are shown in panel F, bars approximate 50 μm. In graphs, data points shown represent actual values for each individual mouse and data are combined from 2 to 6 independent experiments; bars indicate median values and dashed lines indicate the limit of detection; all statistics shown used the Mann–Whitney U two-tailed test; ns: not significant, * *P* < 0.05, **P < 0.01, ****P < 0.0001. See also Fig. S3.

**Fig. 7:**
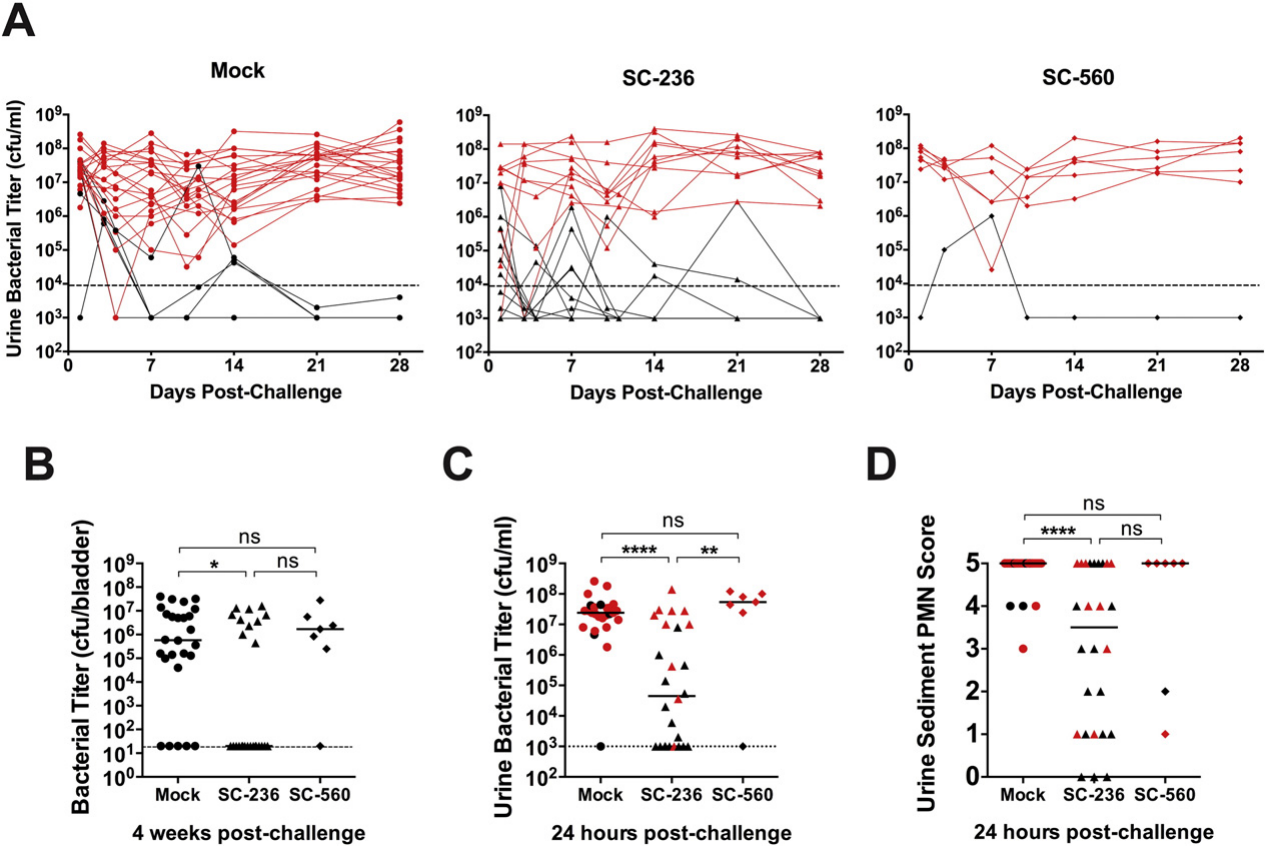
COX-2 inhibition protects sensitized mice against recurrent acute and chronic cystitis. Convalescent mice previously sensitized by a history of chronic cystitis lasting 4 weeks prior to antibiotic treatment were orally gavaged with 10 mg/kg of either a COX-2 specific inhibitor, (SC-236) or a COX-1 specific inhibitor (SC-560), or vehicle alone (Mock) 30 min prior to challenge bladder infection with 10^8^ cfu of the UPEC strain UTI89. SC-560 treatments were repeated at 8 hpi, due to its short half-life. (A) Longitudinal urinalysis for bacterial enumeration was performed over 4 weeks of challenge infection. (B) Bladder titers were determined at 4 weeks post-challenge. (C) Urine bacterial titers and (D) the abundance of neutrophils in the urine sediment were quantitated at 24 h post-challenge. Red data points in panels A, C and D indicate urine parameters for mice that had bladder titers > 10^4^ cfu at sacrifice in panel B. In graphs, data points shown represent actual values for each individual mouse and data are combined from 3 independent experiments; bars indicate median values and dashed lines indicate the limit of detection; all statistics shown used the Mann–Whitney U two-tailed test; ns: not significant, * *P* < 0.05, **P < 0.01, ****P < 0.0001.

To determine whether COX-2 activity alters the susceptibility of C3H/HeN mice to severe acute, chronic and recurrent cystitis, we pretreated C3H/HeN mice orally with a non-selective COX inhibitor (indomethacin), a COX-2 specific inhibitor (SC-236), or a COX-1 specific inhibitor (SC-560). To block COX activity during the first 24 h of infection we dosed mice again with indomethacin and SC-560 at 8 hpi, due to their short half-lives, but not with SC-236, which has a reported half-life of several days (Teng et al., 2003, Penning et al., 1997). COX-2 but not COX-1 inhibition significantly reduced the severity of pyuria at 24 hpi (Fig. 6A) and reduced the incidence of chronic cystitis (Fig. 6B). The decreased severity of infection was independent of the early acute bacterial load, as SC-236 treatment did not change the number of IBCs formed in the bladder at 6 hpi (Fig. S3A). However, bladder bacterial titers were significantly lower in SC-236 treated mice at 24 hpi (Fig. S3B), suggesting that bacterial clearance was facilitated by blockade of COX-2. The enhanced bacterial clearance is unlikely a consequence of direct antimicrobial activity against UPEC, as concentrations of SC-236 up to 200 μg/ml in broth did not affect the growth of UTI89 in vitro (data not shown). Surprisingly, SC-236 treatment did not alter myeloid cell recruitment to the infected bladder (Fig. 6C). Yet, gross bladder inflammation was significantly reduced, as evidenced by less bladder edema (Fig. 6D) and urothelial erosion (Fig. 6E and F) in treated bladders. Urothelial exfoliation was not visibly altered. Finally, in our model of rUTI, pretreatment of sensitized mice with the COX-2 specific inhibitor, SC-236, prior to high dose (10^8^ cfu) UPEC challenge significantly decreased the incidence of recurrent chronic cystitis compared to treatment with vehicle or the COX-1 specific inhibitor SC-560 (Fig. 7A and B, 31% (8/26) vs. 77% (20/26) and 86% (6/7), respectively, *P* < 0.05 for each, Fisher's exact test). Strikingly, the majority of the SC-236 pretreated mice had urine titers at or near the limit of detection for all or most of the experiment (Fig. 7A) and the median urine bacterial load at 24 h post-challenge was reduced nearly 1000-fold by COX-2 inhibition (Fig. 7C). Furthermore, as in naïve mice, SC-236 pretreatment of sensitized mice resulted in a dramatic reduction in neutrophil infiltration of the urine at 24 h post-challenge (Fig. 7D). These data suggest that inhibition of COX-2 protects against severe acute, chronic, and recurrent infection by preventing urothelial transmigration by neutrophils and harmful damage to the urothelial barrier, while allowing beneficial innate responses, such as immune cell recruitment and exfoliation of infected superficial cells.

## 4 Discussion

The rapid emergence of multidrug resistant UPEC clones, including strains that express carbapenemases, highlights the urgent need for alternatives for treatment and prevention of rUTI. Here, we provide evidence that high levels of neutrophil-mediated damage within the urothelial barrier contribute to host susceptibility to rUTI. Neutrophil-mediated mucosal damage has been demonstrated to exacerbate infection in other models, including both influenza and group A streptococcal infections of the respiratory tract (Brandes et al., 2013, Bhowmick et al., 2013). Consistent with this, we identified increased serum levels of chemokines and growth factors involved in myeloid cell inflammation during an initial UTI as biomarkers for increased susceptibility to rUTI within three months, particularly in patients for whom the initial UTI was their first lifetime UTI. We found that inflammatory monocyte recruitment to the infected bladder was not absolutely necessary for urothelial transmigration by neutrophils in contrast to results in a recent study of UTI using C57BL/6J mice (Schiwon et al., 2014). Our data suggest that bladder resident macrophage responses and COX-2 signaling compensate for the loss of inflammatory monocyte recruitment in C3H/HeN mice. Further, our proteomics study demonstrated a global reorganization of urothelial markers in convalescent mice with a history of chronic cystitis (“sensitized”). This apparent mucosal remodeling is consistent with our hypothesis that upon bacterial challenge the bladders of sensitized mice are prone to more severe inflammation and mucosal damage from proteases secreted by neutrophils and other immune cells. Overall, these findings set the stage for targeted translational studies of these proteins in clinical specimens.

Importantly, we provide mechanistic rationale for the use of non-steroidal anti-inflammatory drugs (NSAIDs) or COX-2 inhibitors such as celecoxib in the treatment and prevention of UTI in susceptible patients. We found that inhibition of COX-2 in mice reduced pyuria and prevented mucosal damage, but did not disrupt known beneficial mucosal responses, such as urothelial exfoliation and overall immune cell recruitment to the bladder (Ingersoll et al., 2008, Mulvey et al., 1998). This may explain the results of a small clinical trial that compared ibuprofen to the fluoroquinolone antibiotic ciprofloxacin in a 3-day course of treatment for UPEC UTI, and found similar improvement in clinical outcome at days 4 and 7 after initiation of therapy (Bleidorn et al., 2010). Although larger clinical trials are needed, together our studies suggest that NSAIDs do not just mask symptoms of UTI, but also affect the clinical outcome suggesting that these readily available and safe therapies could replace the use of antibiotic prophylaxis in susceptible individuals during periods of high risk, e.g. periods of sexual activity in women with coital-associated rUTI. The COX-2 inhibitor celecoxib has also been reported to enhance the sensitivity of multidrug resistant bacteria to antibiotics, providing a dual rationale for the use of this drug in conjunction with antibiotic therapy (Kalle and Rizvi, 2011).

The following are the supplementary data related to this article.

Supplementary material.

Table S2 Identified and quantified proteins from the proteomic analyses.

## Author Contributions

T.J.H., T.S.S., G.C.H., W.F.S., M.C. and S.J.H. designed the experiments. A.E.S. and T.M.H. designed and supervised the clinical study. P.L.R. performed the statistical analysis of the clinical data. T.J.H., J.M.B. and D.J.S. performed the in vivo experiments. T.J.H. and J.M.B. extracted RNA and performed qRT-PCR. T.J.H. performed histopathological analysis. T.J.H. and T.E.R. performed immunofluorescence microscopy. S.vdP. performed proteomics experiments. H.M. performed in situ hybridization for *Ptgs2*. M.M. provided the anti-CCR2 monoclonal antibody. R.A.S. provided clodronate and PBS encapsulated liposomes. T.J.H., S.vdP., J.M.B., and D.J.S. analyzed data. T.J.H. and S.J.H. wrote the manuscript incorporating all of the authors' editorial input.

## Figures and Tables

**Table 1:** Description of women at enrollment (N = 326).

Characteristic	Value
Age in years, median (range)	21 (18–45)
Never married, n (%)	272 (83)
Race, n (%)	
White	218 (67)
Asian	65 (20)
African American	4 (1)
Other	39 (12)
Hispanic, n (%)	20 (6)
UTI history	
UTI, ever, n (%)	248 (76)
≥ 3 UTIs in lifetime, n (%)	153 (47)
Pyelonephritis, ever, n (%)	32 (10)
UTI past year, n (%)	167 (51)
Sexual activity, past month	
Sexually active, n (%)	315 (97)
Vaginal intercourse episodes, median (range)	8 (0–75)
UTI type (enrollment), n (%)	
*E. coli*	249 (76)
Gram negative, not *E. coli*	23 (7)
Not Gram negative	28 (9)
Culture negative	26 (8)

**Table 2:** Serum cytokines and growth factors associated with myeloid cell development and chemotaxis are elevated in patients that subsequently develop recurrent UTI.

Cytokine	rUTI (n = 41)	No-rUTI (n = 45)	Ratio of medians (rUTI/no-rUTI)	*P* value^a^	No prior history of cystitis		Prior history of cystitis	
rUTI (n = 9)	No-rUTI (n = 10)	rUTI (n = 32)	No-rUTI (n = 35)
M-CSF (CSF1)	33.9	25.1	1.4	0.020	46.65	23.01	31.85	26.51
IL-8 (CXCL8)	117.3	59.2	2.0	0.054	125.10	40.29	107.97	62.40
GROa (CXCL1)	156.4	115.5	1.4	0.054	199.70	125.12	146.06	113.99
IL-3	24.2	7.6	3.2	0.084	24.15	9.13	23.93	6.08
MCP-1 (CCL2)	47.1	40.2	1.2	0.098	69.68	36.13	46.55	44.16

All cytokines are reported as median pg/ml serum values.

See also Table S1.

a Statistical analysis performed using Mann–Whitney U two-tailed test.
