# Supplementary material for: Inhibition of Cyclooxygenase-2 Prevents Chronic and Recurrent Cystitis
Source: eBioMedicine. 2014 Oct 24;1(1):46–57. doi: 10.1016/j.ebiom.2014.10.011 (PMC4457352; doi:10.1016/j.ebiom.2014.10.011)
Supplement: Supplementary file 1 — Supplementary material. [file mmc1.pdf]

Supplemental Information

Supplemental Data

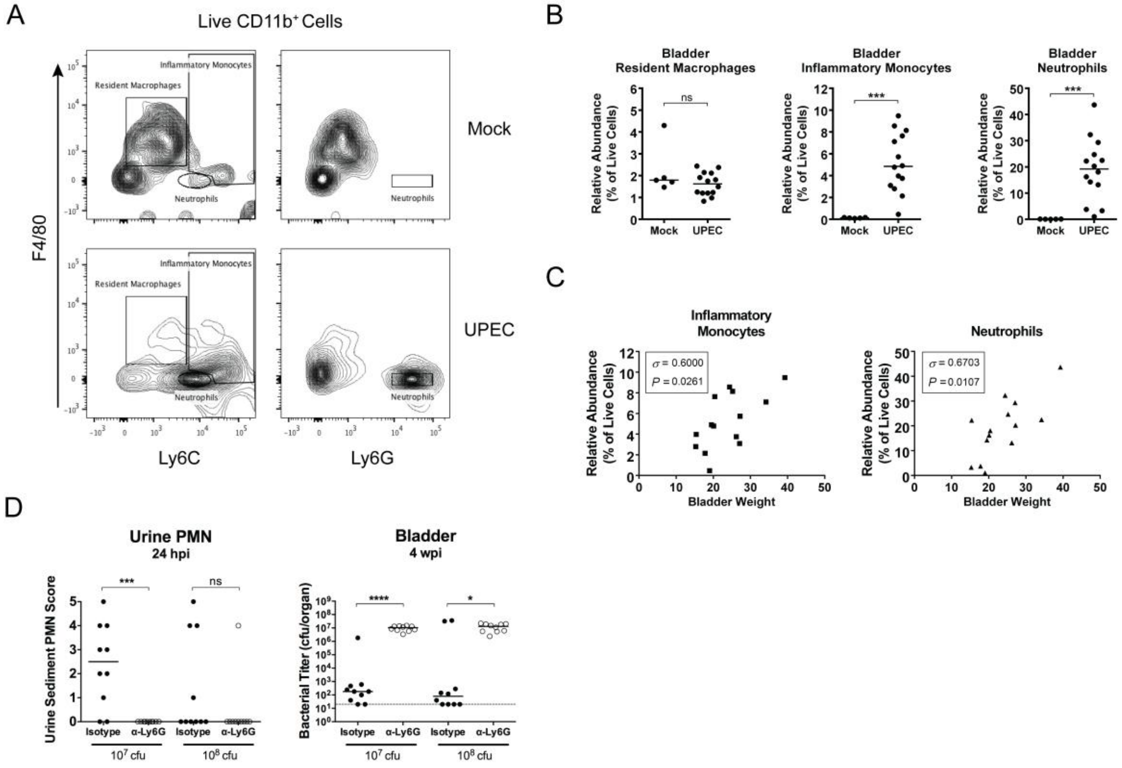

Figure S1. Neutrophils are required to control UPEC infection of the urinary bladder and the magnitude of myeloid cell infiltration into the UPEC infected urinary bladder correlates with the severity of acute inflammation, related to Figure 1. (A-C) Mice were infected with 10^7 cfu UPEC and sacrificed at 24 hpi for analysis of bladder inflammation. Data are representative of two independent experiments. **(A)** Gating strategy to identify bladder resident macrophages, inflammatory monocytes and neutrophils. **(B)** The relative abundances of the indicated cell lineages were determined by flow cytometry. Statistics shown used the Mann-Whitney U two-tailed test; ns: not significant, ***P < 0.001. **(C)** Scatterplot analysis using Spearman's rank-order correlation demonstrating that the magnitude of myeloid cell recruitment to the infected bladder is a strong determinant of bladder weight, an indicator of bladder edema and overall bladder inflammation, at 24 hpi. **(D)** Mice were treated with either 200 µg of the 1A8 anti-Ly6G monoclonal antibody (*1A8*, open circles) or 200 µg of isotype control antibody (*Iso*, closed circles) intraperitoneally 72 and 24 hours prior to intravesical inoculation with UPEC. Data are combined from 2 independent experiments. In graphs, bars indicate median values and dashed lines indicate the limit of detection; all statistics shown used the Mann-Whitney U two-tailed test; ns: not significant, * P < 0.05, ***P < 0.001, ****P < 0.0001.

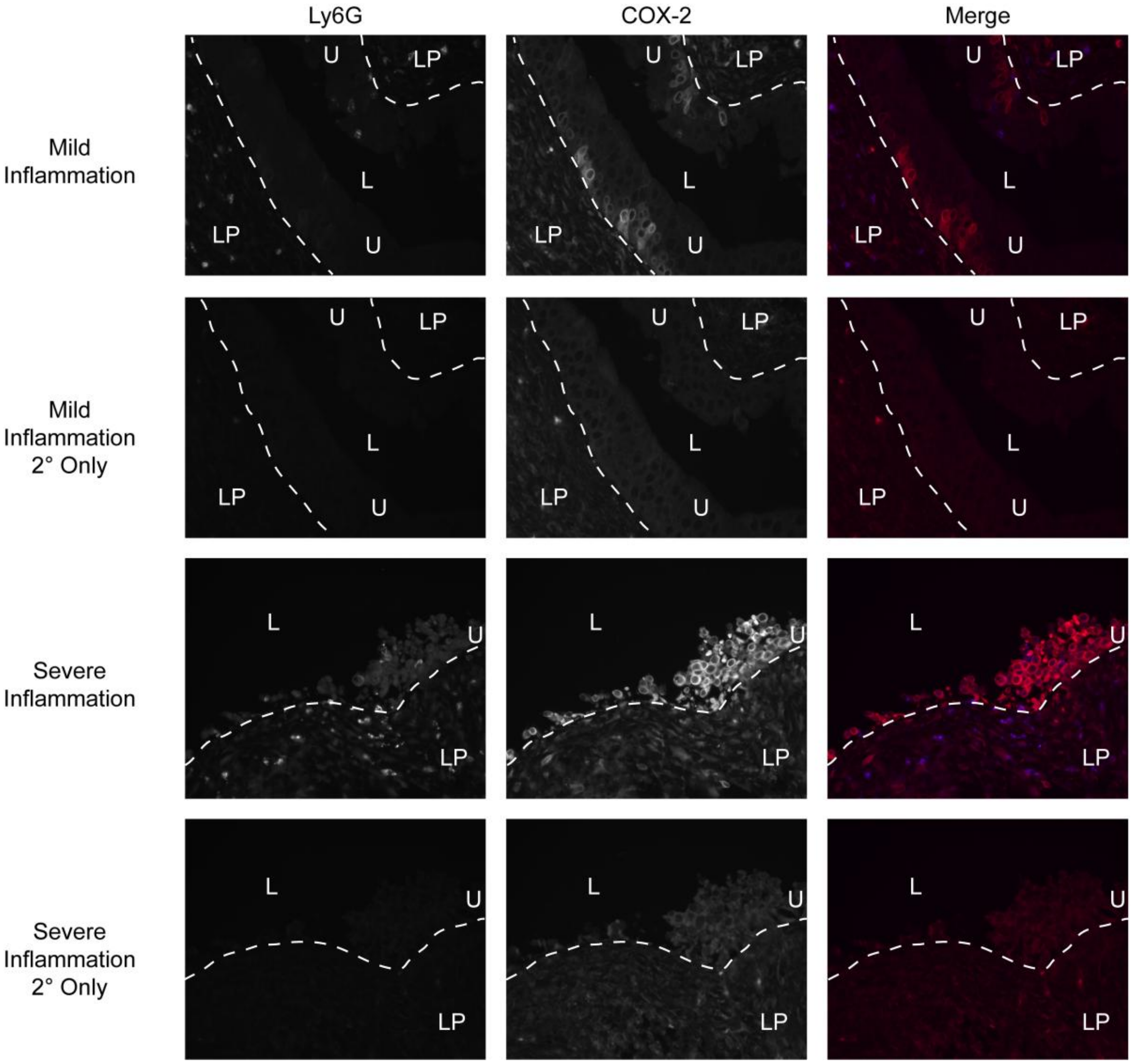

Figure S2. COX-2 immunoreactivity is primarily found in epithelial cells within severely inflamed bladders, related to Figure 5. Specific immunoreactivity of primary antibodies for COX-2 (mouse monoclonal) and Ly6G (rat monoclonal) was determined in fixed, paraffin-embedded sections of UPEC-infected bladders at 24 hpi. Mildly inflamed bladder had an inflammatory score of 2, whereas the severely inflamed bladder had an inflammatory score of 5. "2° only" indicates serial sections that were not stained with primary antibodies to determine non-specific staining attributable to the anti-mouse and anti-rat secondary antibodies. L indicates bladder lumen, U indicates urothelium, LP indicates lamina propria, and dashed line denotes the approximate location of the urothelial basement membrane.

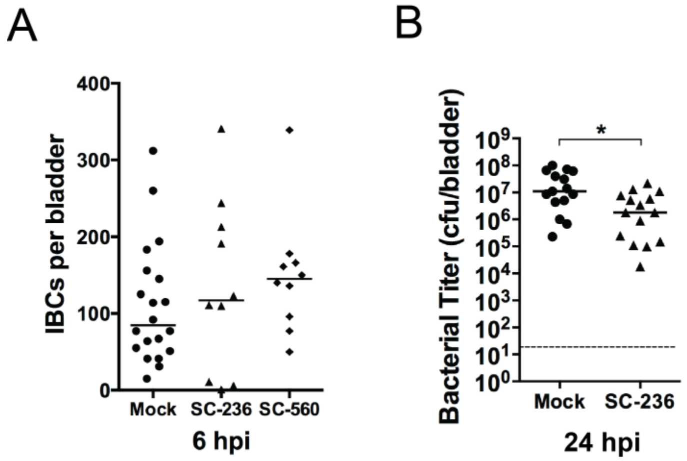

Figure S3. Treatment with a COX-2 inhibitor does not alter early IBC formation, but enhances bacterial clearance by 24 hpi, related to Figure 6. Mice were orally gavaged with 200 μg (10 mg/kg) of either a COX-2 specific inhibitor, (*SC-236*), or a COX-1 specific inhibitor (*SC-560*), or vehicle alone (*Mock*) 30 minutes prior to intravesical inoculation with 10^8 cfu of the UPEC strain UTI89. **(A)** Bladder IBC formation was assayed by LacZ staining at 6 hpi. **(B)** Bladder bacterial titers were enumerated at 24 hpi. In graphs, data points shown represent actual values for each individual mouse and data are combined from 2-4 independent experiments; bars indicate median values and dashed lines indicate the limit of detection; all statistics shown used the Mann-Whitney U two-tailed test; ns: not significant, * $P < 0.05$.

Supplemental Table 1. Serum cytokines associated with the development of rUTI in college-aged women.

V0 Human Sera	rUTI vs. no rUTI			No rUTI (n=45)		rUTI (n=41)	
Cytokine	*P* value[†]	Ratio Means	Ratio Medians	Mean (pg/ml)	Median (pg/ml)	Mean (pg/ml)	Median (pg/ml)
M-CSF (CSF1)	0.020	1.3	1.4	27.5	25.1	36.4	33.9
IL-8 (CXCL8)	0.054	1.9	2.0	106.8	59.2	203.8	117.3
GROa (CXCL1)	0.054	1.1	1.4	137.2	115.5	157.7	156.4
IL-3	0.084	1.4	3.2	19.9	7.6	27.0	24.2
MCP-1 (CCL2)	0.098	1.2	1.2	47.0	40.2	57.8	47.1
IL-16	0.100	1.4	1.1	155.0	135.6	214.6	145.6
TNF-b (LTa)	0.113	1.3	1.2	4.2	4.1	5.4	5.2
RANTES (CCL5)	0.115	1.1	1.2	3463.1	3349.1	3804.5	3905.0
NGF	0.119	1.2	1.1	1.9	1.9	2.2	2.1
IL-4	0.121	1.1	1.1	10.5	10.6	11.2	11.2
Ifn-α2	0.138	1.2	1.1	19.9	22.3	23.2	24.2
IL-1a	0.148	1.3	1.2	1.9	1.7	2.5	2.0
TRAIL	0.226	1.1	1.2	84.4	80.0	92.8	94.7
MIF	0.252	1.2	1.2	3710.0	2946.3	4626.9	3639.1
CTACK (CCL27)	0.259	0.9	0.9	608.3	610.8	572.1	543.8
IL-10	0.292	1.1	1.2	4.3	3.9	4.5	4.5
HGF	0.300	1.2	1.1	595.9	523.8	687.4	599.4
LIF	0.303	1.2	1.2	12.3	11.1	14.3	13.0
VEGF	0.355	1.0	1.2	179.6	142.2	184.6	173.0
IL-12p70	0.371	1.0	1.2	33.4	28.6	34.1	33.5
IL-1b	0.375	1.3	1.0	2.2	2.2	2.8	2.3
PDGF-bb	0.383	1.0	1.1	6817.4	6374.5	7010.4	7031.9
IL-12p40	0.398	1.1	1.2	158.0	149.8	176.6	176.4
SCF	0.431	0.9	1.0	131.2	126.7	122.8	126.8
IL-2	0.468	1.4	1.1	7.5	6.7	10.4	7.1
MIG (CXCL9)	0.542	1.4	1.1	866.3	709.9	1248.5	759.1
MCP-3 (CCL7)	0.545	1.2	1.2	25.4	13.6	29.4	16.5
IL-5	0.571	1.0	1.1	3.2	2.9	3.3	3.2
Eotaxin	0.571	1.1	1.0	79.0	73.5	84.9	74.9
GM-CSF (CSF2)	0.619	1.1	1.2	32.8	26.3	36.8	31.6
IL-7	0.644	1.0	1.0	10.7	10.1	10.3	10.5
IL-18	0.697	1.0	1.1	94.2	84.0	95.9	90.4
IL-1ra	0.729	1.0	1.0	274.0	256.9	273.4	266.1
SDF-1a (CXCL12a)	0.746	1.0	1.0	91.1	90.1	91.0	88.7
SCGF-b (CLEC11a)	0.759	1.0	1.0	27807.4	26872.1	29147.5	26439.0
IL-13	0.766	1.0	1.0	10.4	10.1	10.3	10.1

IL-2ra	0.766	1.0	1.1	114.4	104.8	113.8	115.9
IL-6	0.815	1.2	1.0	14.1	13.3	16.6	12.8
IP-10 (CXCL10)	0.826	1.3	1.0	716.5	594.4	910.8	611.2
IL-15	0.832	1.2	0.9	7.9	7.2	9.7	6.3
IL-9	0.859	1.2	1.0	61.2	37.0	71.1	38.1
Ifn-γ	0.883	1.2	1.0	116.2	112.7	141.8	114.3
G-CSF (CSF3)	0.897	1.0	1.0	33.7	34.1	34.8	33.3
MIP-1b	0.907	1.0	1.0	139.2	127.8	132.9	130.2
FGF-basic	0.914	1.1	1.0	28.1	27.2	29.7	26.4
IL-17	0.979	1.0	1.0	54.0	52.9	53.2	53.0
TNF-a	0.979	1.3	1.0	33.3	31.8	43.3	31.2
MIP-1a (CCL3)	0.983	1.0	1.0	6.3	6.3	6.4	6.1

† *P* value determined by two-tailed Mann-Whitney U test
